# We should do better in accounting for multiple births in neonatal randomised trials: a methodological systematic review

**DOI:** 10.1136/archdischild-2024-327983

**Published:** 2024-12-09

**Authors:** Kristy P Robledo, Sol Libesman, Lisa Nicole Yelland

**Affiliations:** 1 NHMRC Clinical Trials Centre, The University of Sydney, Sydney, New South Wales, Australia; 2 Women and Kids Theme, South Australian Health and Medical Research Institute Limited, Adelaide, South Australia, Australia; 3 School of Public Health, The University of Adelaide, Adelaide, South Australia, Australia

**Keywords:** Statistics, Twins, Neonatology, Paediatrics

## Abstract

**Objective:**

To conduct a methodological systematic review of multicentre trials of premature infants to (1) determine if and how multiple births have been considered in the design, analysis and reporting of recent trials and (2) assess whether there has been an improvement since the last review was conducted 10 years ago.

**Design:**

A systematic search was conducted in PubMed on 28 June 2023 for articles published between June 2018 and June 2023. Articles were eligible for inclusion if they were a multicentre randomised trial of infants born preterm and reported the results of a primary outcome that was measured on an infant or could be attributed to an infant.

**Results:**

We reviewed 62/74 trials (80%), after determining it was unclear if multiple births were present in the other 20%. 87% of trials (54/62) did not account for multiple births in their sample size calculations and 48% (30/62) did not account for clustering due to multiple births in their analyses. Problems were not limited to lower-ranked journals. No trials reported the intraclass correlation coefficient for any outcomes, indicating the degree of clustering present.

**Conclusions:**

Persistent problems remain with the design and analysis of multicentre trials of premature infants due to ignoring the complexity that comes with the inclusion of multiple births, despite methods available to address this. Trialists should consider the impact of multiple births in their trial design and analysis. Readers of neonatal trials should be aware of these issues, particularly those who peer review papers.

**WHAT IS ALREADY KNOWN ON THIS TOPIC**

- Infants from a multiple birth generally provide less information about the effect of an intervention than unrelated singletons, due to similarities (correlation) in their outcomes. Earlier reviews from over 10 years ago have shown that clinical trials of premature infants often ignore multiple births, impacting on a trial’s ability to draw accurate conclusions about treatment differences.

**WHAT THIS STUDY ADDS**

- In this methodological systematic review, 87% of trials (54/62) did not account for multiple births in their sample size calculations and 48% (30/62) did not account for clustering due to multiple births in their analyses.

**HOW THIS STUDY MIGHT AFFECT RESEARCH, PRACTICE OR POLICY**

- Our review will raise awareness of the importance of accounting for multiple births in the trial design and analysis among trialists and journal reviewers.

## Introduction

 Premature birth complication is the leading cause of death for neonates.^1^ Almost half of under-five deaths in 2021 occurred in the neonatal period,^2^ the first month of life, and reducing this mortality rate is a global priority in the Sustainable Development Goals.^3^ Numerous clinical trials are undertaken each year aimed at reducing mortality or negative health consequences of preterm birth; however, only some of these trials include infants from multiple births. Over 50% of multiple gestation births are preterm,^4^ and hence the decision to include or exclude these infants from a trial has an important impact on the generalisability of the trial results.

When multiple births are included in a trial, this complicates both the statistical analyses and the sample size calculations. This complexity arises from the correlation between outcomes of infants from the same birth mother, driven by shared environmental and genetic factors.^5 6^ In analyses, a set of twins generally provides less information about the effect of an intervention than two unrelated singletons. Ignoring this correlation or clustering due to multiple births in analyses can inflate type I error rates,^7^ leading to ineffective treatments being more likely to be recommended for practice. Analytical methods to take into account this correlation, such as generalised estimating equations^8^ or mixed effects models,^9 10^ are widely discussed and available in common software packages.

When performing sample size calculations, ignoring the impact of multiple births on these calculations will often result in an underpowered trial. Despite this, and the high prevalence of multiple births in preterm populations, two earlier reviews demonstrated that only 1 out of 26 trials took multiple births into account in the sample size calculations^11^ and clustering due to multiple births is commonly ignored in analyses.^11 12^

10 years have passed since the last review was conducted, and it is unclear whether these poor analytical practices have improved, or whether new methods allowing for the correlation between outcomes of multiple births in the sample size calculation^13 14^ are being used in practice. The aims of this methodological systematic review were to (1) determine if and how multiple births have been considered in the design, analysis and reporting of recent multicentre trials involving preterm infants and (2) assess whether there has been an improvement since the last review was conducted 10 years ago.^11^

## Methods

This methodological systematic review was conducted according to a prespecified protocol (online supplemental material 1). Consistently with the previous review on this topic,^11^ we focused on preterm infants, where the definition of preterm was left to the individual trial, as multiple births are more common among preterm births. We also focused on multicentre trials as these are generally larger and involve collaboration between trialists and statisticians, who may be more attuned to the design and analysis implications when including multiple births. The search was conducted in PubMed on 28 June 2023, using the search terms “(preterm) OR (prematur*)” and “(multi-cent*) or (multicent*)”, and applying the filters for “Randomized controlled trial” and “Published within the past 5 years”. The inclusion of trials within the past 5 years (2018–2023) was to ensure a lag following the previous publication in 2015,^11^ to assess improvements.

Articles were eligible for inclusion if they were a multicentre trial (including two or more sites) and reported the results of a primary outcome that was measured on an infant or could be attributed to an infant. The primary outcome was identified by the authors as the primary outcome, or the outcome that was used to determine the sample size if none was named (or the first outcome if multiple were listed). Articles were excluded if the trial was listed as a pilot, phase I or phase II trial, if they reported the protocol or the statistical analysis plan only, if they reported either the follow-up of an earlier trial, secondary analyses or analyses of multiple trials combined (including meta-analyses). However, if an eligible randomised controlled trial also reported a meta-analysis, the reported randomised controlled trial was eligible for inclusion. No automated tools were used to screen or assess eligibility.

Titles and abstracts of all identified articles were examined by one reviewer (KPR) to determine eligibility for this review. If eligible, data regarding trial design and analyses (see online supplemental material 1) were extracted from the full text (including any information within each paper’s supplementary material(s) into a purpose-built REDCap database. The SCImago Journal Ranking (SJR 2023), which is a ranking measure of a journal’s citation potential (1=average citation potential), was used (continuously) as a proxy for the journal quality, noting that this is only one contributor to quality. A 10% stratified random sample (56 articles: 28 eligible and 28 ineligible) was selected for a second review and data extraction by SL. No major discrepancies were identified in this sample, and given no formal meta-analyses were planned, this level of checking and agreement was considered satisfactory to the study team.

All analyses were performed in R V.4.1.2.^15^ Data were summarised descriptively using proportions, medians and boxplots.

## Results

**Figure 1:**
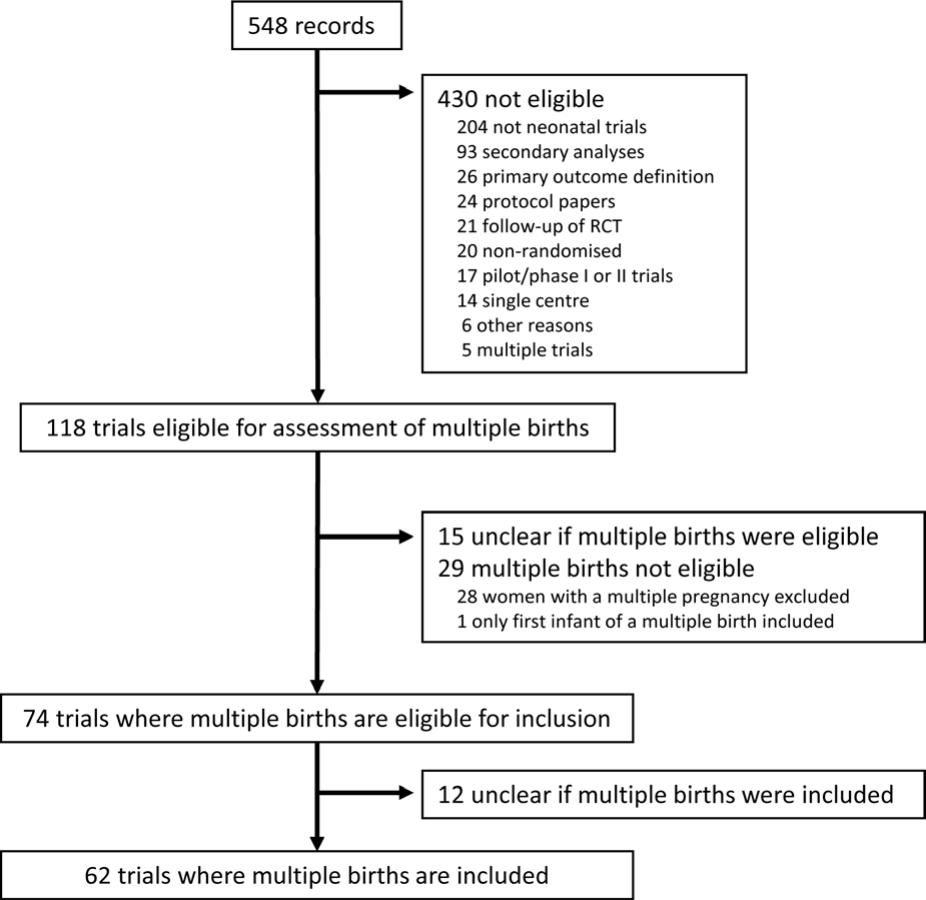
Flow chart from the main search to the trials included in this methodological review. RCT, randomised controlled trial.

A total of 548 records were identified in the search, of which 430 were not eligible (figure 1). Common reasons for ineligibility were they did not report a neonatal trial (n=204) or they reported secondary analyses (n=93). This left 118 publications available for evaluating whether multiple births were included.

### Eligibility and inclusion of multiple births

**Figure 2:**
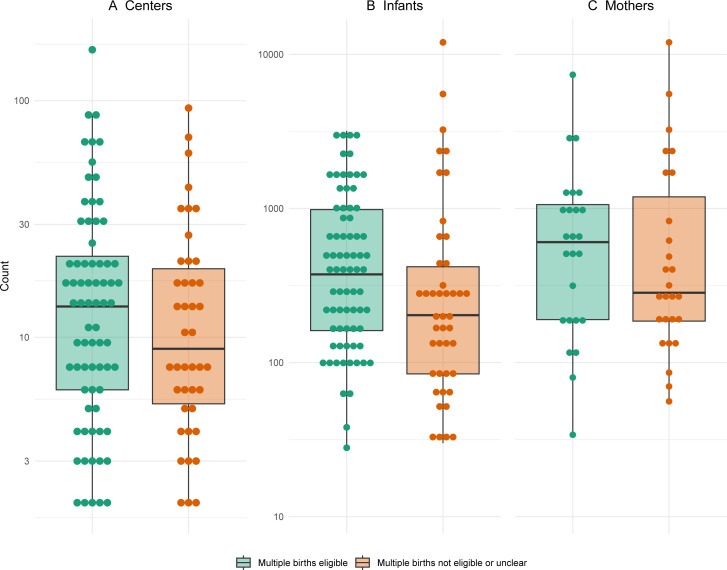
Comparison of trial size between those trials with multiple births eligible for inclusion (n=74) and trials where multiple births were ineligible, or their eligibility was unclear (n=44). Size is presented as the number of study centres (**A**), infants (**B**) and women (**C**). Counts are given on the log scale for readability, and each dot corresponds to a trial.

Out of 118 trials available for assessment of multiple births, 25% (29/118) listed multiple births as ineligible and 13% (15/118) did not report information regarding the eligibility of multiple births in the inclusion and exclusion criteria. Additionally, there was no mention of multiple births in any text, tables, figures or supplementary materials. This left 63% (74/118) where multiple births were eligible for inclusion. Trials that included multiple births as eligible were larger and included more study centres (median 14 vs 9), infants (median 374 vs 203) and women (median 605 vs 284) compared with those trials that excluded multiple births or were unclear (figure 2).

Of the 74 trials where multiple births were eligible for inclusion, 16% (12/74) were unclear in text, figures and tables as to whether any infants from multiple births were randomised into the trial. This left 62 multicentre trials where multiple births were both eligible and included.

### Design features of trials including multiple births

Infants from multiple births made up around a quarter of the trial participants (median 26%, Q1–Q3: 19–31%) in those 62 trials that included multiple births. This information was reported by the treatment group in 97% of trials (60/62), allowing readers to assess balance. In 50% of trials, the highest order of multiple births recruited was unclear, reported only as ‘multiple birth’ (31/62, table 1). Infants were most commonly randomised (61%; 38/62), followed by mothers (35%; 22/62) and sites (3%; 2/62). In the 38 trials that randomised the infant to the intervention, 24% (9/38) randomised multiple births to the same intervention, 35% (13/38) randomised infants from the same birth independently, and 41% (15/38) were unclear in how infants from the same birth were randomised. None of the trials that either randomised mothers or assigned infants from multiple births to the same intervention identified themselves as cluster randomised.

**Table 1:** Design characteristics of neonatal trials that included multiple births (n=62)

Characteristic	N=62*
Proportion of women with multiple births, %	9 (2, 28)
Unknown	40 (65%)
Proportion of infants from a multiple birth, %	26 (19, 31)
Unknown	3 (5%)
Reported information enabling assessment of balance of multiples between groups	
No	2 (3%)
Yes	60 (97%)
Highest order multiples included in the trial	
Twins	23 (37%)
Triplets	6 (10%)
Higher order multiples	2 (3%)
Unclear	31 (50%)
Who was randomised	
Site was randomised	2 (3%)
Mother (cluster randomised†)	22 (35%)
Infant	38 (61%)
If infant was randomised, how were multiples randomised	
Infants randomised independently	14 (37%)
Randomised to same group (cluster randomised†)	9 (24%)
Unclear	15 (39%)
(Infant not randomised)	24
Method of randomisation	
Stratified blocks (covariate-adaptive randomisation‡)	39 (63%)
Minimisation (covariate-adaptive randomisation‡)	9 (15%)
Simple	5 (8%)
Other§	7 (11%)
Unclear	2 (3%)
If covariate-adaptive randomisation‡ was used, was multiple births a balancing factor	
No	38 (79%)
Yes	10 (21%)
(Covariate-adaptive randomisation not used)	14
Who received the intervention	
Mother	22 (35%)
Infant	39 (63%)
Both infant and mother	1 (1.6%)
On what level is the outcome defined	
Cluster level	11 (18%)
Infant level	51 (82%)
Did the sample size calculation account for clustering due to multiples	
No	54 (87%)
Yes	8 (13%)

* Median (IQR); n (%).

† Indicates cluster randomised trials (n=31).

‡ Indicates covariate balancing method was used (n=48).

§ Other randomisation methods include unstratified blocks and stratified random number lists (with no blocking).

Randomisation was generally performed to balance on baseline covariates, either by stratified permuted blocks (63%; 39/62) or minimisation (15%; 9/62). Multiple births were used as a balancing factor in 21% of these trials (10/48).

Most trials assessed an intervention that the infant received (63%; 39/62) rather than the mother, and the primary outcome was measured on the infant level (82%; 51/62). When measured on the mother (cluster level), the outcome was defined as *any* occurrence of the outcome over all the infants from the multiple birth and therefore could have been defined instead at the infant level. The primary outcome for these 62 trials is given in online supplemental material 2, table 1.

**Figure 3:**
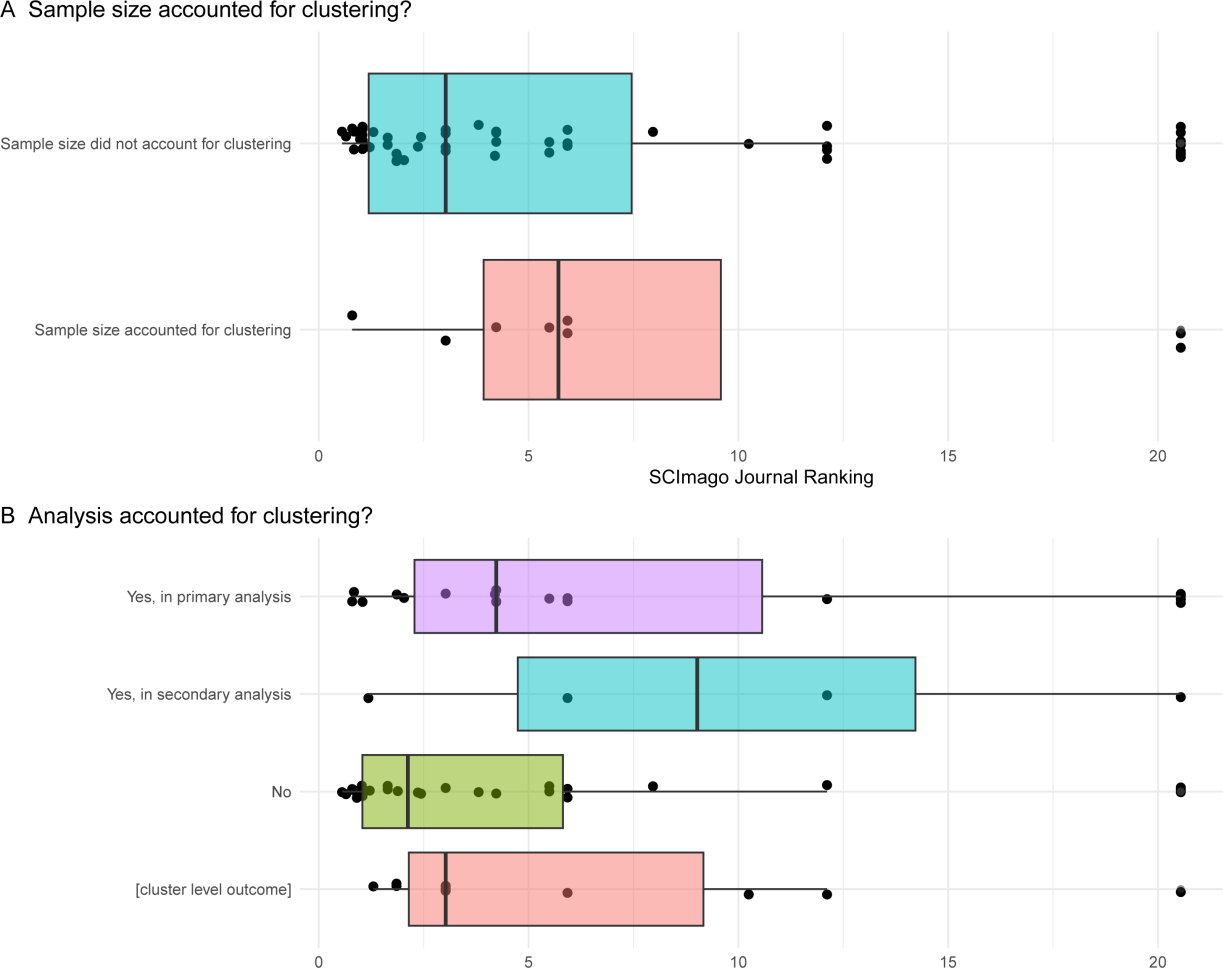
Comparison of the SCImago Journal Ranking (SJR 2023) of the journals in which the trials were published, between those that accounted for clustering in (**A**) the sample size calculations or (**B**) the analysis. Each dot corresponds to one of the 62 trials, and each trial appears in each panel.

The vast majority of trials (54/62, including 42/51 with an infant-level primary outcome) did not consider the impact of the inclusion of multiple births (ie, the clustering or the correlation) in the sample size calculation, and this problem was not limited to journals with lower or higher rankings (figure 3A). No reason for this was given in any of these trials. In those eight that did account for clustering, the details of how they achieved this were inconsistently and often incompletely reported. One trial simply stated that the sample was inflated to ‘to account for the impact of twins’.^16^ Two trials reported the expected prevalence of multiple births (reporting prevalence of 25%^17 18^), and two reported the assumed average cluster size (1.25^18^ and 2^19^). Four trials stated the assumed value of the intraclass correlation coefficient (ICC) as a measure of the degree of expected similarity of outcomes of infants from the same birth, which ranged between 0.3 and 0.9; however, only two provided a reference for this value.^17 20^ Five trials explicitly reported a design effect that fell in the range of 1.1–1.2, indicating the required sample size was 10–20% larger than a trial including singletons only.

### Analyses of trials including multiple births

Multiple births were considered in analyses in a variety of ways. Subgroups according to an infant’s multiple birth status were formally tested in 10% of trials (6/62), and the treatment effect was reported within each subgroup in 13% of trials (8/62). Adjusting models for an infant’s multiple birth status (as a fixed effect) was performed in 23% of trials (14/62). When considering the 10 trials that included multiple births as a balancing factor in the randomisation, 9 of these (90%) adjusted for multiple birth status.

Almost 50% of trials did not account for the clustering due to multiple births in any analyses (30/62), including some published in higher-ranking journals (figure 3B). Only one of these 30 trials provided a rationale,^21^ namely that only two sets of twins were randomised. When clustering was taken into account (35%; 22/62), this was more commonly in the primary analysis (18/22) rather than in a secondary or sensitivity analysis (4/22). Accounting for clustering was performed by generalised estimating equations (GEE) in 55% (12/22), mixed models in 27% (6/22), both GEE and mixed models in one trial, modified Poisson or logistic regression with robust SE estimates in two trials, and one trial stated in their supplementary material that the analysis model included ‘a clustering feature for multiple births’. In those trials that performed analyses by GEE (n=13), only three reported the working correlation structure used (all reported an exchangeable working correlation structure). All trials that accounted for clustering in the sample size calculations also accounted for clustering in the primary analysis (table 2).

**Table 2:** Analysis methods of neonatal trials that included multiple births (n=62), by whether the sample size accounted for multiple births or not

Characteristic	Sample size accounted for clustering, N=8	Sample size did not account for clustering, N=54	Overall, N=62
Interaction p value given for treatment × multiple birth effect	0 (0%)	6 (11%)	6 (10%)
Treatment effect reported in multiple births as a subgroup	0 (0%)	8 (15%)	8 (13%)
Adjusted analysis performed with multiple births as a fixed effect	3 (38%)	11 (20%)	14 (23%)
Did any analysis account for clustering due to multiples			
NA (cluster level outcome)	0 (0%)	10 (19%)	10 (16%)*
Yes, in primary analysis	8 (100%)	10 (19%)	18 (29%)
Yes, in secondary/sensitivity analysis	0 (0%)	4 (7%)	4 (7%)
No	0 (0%)	30 (56%)	30 (48%)
How was clustering due to multiples considered in the analysis			
GEE	4 (50%)	8 (57%)	12 (55%)
Mixed model	3 (38%)	3 (21%)	6 (27%)
Other	1 (13%)	2 (14%)	3 (14%)
Unclear	0 (0%)	1 (7%)	1 (5%)
(Clustering not considered)	0	40	40
Method of MI			
Chained equations	1 (100%)	5 (83%)	6 (86%)
Unclear	0 (0%)	1 (17%)	1 (14%)
(MI not performed)	7	48	55
MI accounted for the clustering of multiple births			
No	1 (100%)	2 (33%)	3 (43%)
Yes	0 (0%)	2 (33%)	2 (29%)
Unclear	0 (0%)	2 (33%)	2 (29%)
(MI not performed)	7	48	55

All statistics are reported as n (%).

* Although 11 trials had a cluster-level primary outcome, one trial analysed secondary outcomes on an infant level.

GEE, generalised estimating equation; MI, multiple imputation.

Multiple imputation was performed in 11% of trials (7/62), most commonly by chained equations (6/7). Only two trials^22 23^ reported using multiple births (singleton vs multiple births) as a fixed covariate in the imputation models followed by a mixed model or GEE analysis of the imputed datasets, taking into account the clustering of outcomes.

No trials reported the ICC from any analyses. Additionally, no trials used the recent estimand framework^24^ to give a precise statement of the treatment effect of interest. Additional details on trial design and methods used are provided in online supplemental material 2.

## Discussion

In this methodological systematic review, we included 62 multicentre trials conducted in preterm populations that recruited infants from multiple births, published over a recent 5-year period. Our review reveals four key findings. First, 16% of the eligible trials were unable to be included as they did not report if multiple births were eligible or included in the trial; second, 87% of trials did not account for multiple births in their sample size calculations; third, 48% did not account for clustering due to multiple births in their analyses; and finally, no trials reported the ICC for any outcomes to indicate the degree of clustering present in the data and assist with planning future trials. These findings clearly highlight major limitations in the design, analysis and reporting of neonatal trials, that are not restricted to lower-ranking journals and need to be addressed. Authors and readers of neonatal trial papers should be aware of these issues, particularly those that are called on to peer review papers.

It has been 10 years since the last review of how multiple births are handled in randomised trials,^11^ and our work shows that there is still substantial room for improvement. The percentage of trials accounting for multiple births in the sample size calculations has increased somewhat from 4% to 13%, supported by the development of sample size methods and a free calculator specifically for this setting,^14^ though no studies cited this calculator. Despite these resources and the relatively high prevalence of multiple births in trials targeting preterm infants, the vast majority of trials continue to ignore this important issue, and these issues are apparent across all journal rankings. There has been little change in the percentage of trials accounting for clustering due to multiple births in their primary analysis (31% previously,^11^ now 29%), despite reporting guidelines around the importance of accounting for clustering,^25^ and a wealth of literature highlighting the importance in the specific context of multiple births.^5^,^712 26^ Other approaches to account for multiple births in the design and analysis have gained popularity over the past 10 years,^11^ including stratifying by multiple birth status, adjusting for multiple birth status in the analysis as a fixed effect, and testing for different treatment effects between singletons and multiples, although none of these approaches can make up for ignoring the clustering in the design and analysis. More trials used multiple imputation here than in the previous review (11% vs 4%). Recent recommendations indicate that imputation should be performed within singletons and multiple births separately, with imputation for multiple births to be performed in a ‘wide format’ to account for the correlation^31^; however, no trials in our review used this approach.

Ignoring clustering in sample size calculations can result in an underpowered trial. Those trials in the review that did account for multiples inflated their sample size by 10–20%, highlighting the substantial impact multiples can have on the sample size. Trialists can account for multiples when designing future trials using a freely available online calculator.^14^ This requires an estimate of the ICC, which measures the degree of similarity between outcomes of infants from the same birth. We are aware of only one previous study summarising ICCs for common infant outcomes across multiple trials^32^ and note that none of the trials included in our review reported any ICCs. We encourage the reporting of ICCs in individual trials to assist with sample size calculations for future trials, as well as comply with the Consolidated Standards of Reporting Trials (CONSORT) statement for cluster randomised trials.^25^ This guideline holds particular relevance for neonatal trials that either randomise mothers or assign infants from the same birth to the same treatment group, though they may more accurately be described as ‘partially clustered trials’ than cluster randomised trials to reflect the inclusion of single and multiple births.^33^

The continued widespread practice of ignoring clustering due to multiple births in the analysis is somewhat surprising. Methods to account for clustering, such as GEEs and mixed effects models, are readily available in common software packages, and numerous previous studies have highlighted the importance of accounting for clustering due specifically to multiple births in the analysis.^5^,^712 26^ However, the lack of practical guidance and example code may be limiting the use of clustered data methods by trial analysts and represents an important area for future research. Among trials that did account for clustering in the analysis, GEEs were the most popular approach. GEEs have been shown to perform well in the multiple birth setting^7 30^ and provide treatment effects with an infant-level interpretation when the cluster size (ie, number of infants per mother) may be informative, provided an independent working correlation structure is specified.^34^

The recent addendum to the International Council for Harmonisation of Technical Requirements for Registration of Pharmaceuticals for Human Use (ICH) E9 harmonised guideline on statistical principles for clinical trials highlights the importance of clearly and precisely defining the treatment effect of interest that corresponds to the research question of interest, otherwise known as the ‘estimand’.^24^ No trials in our review reported the estimand of interest. This is not surprising, since the estimand framework was published after many trials in our review were designed and/or analysed, though estimands will likely be an important feature in reports of future neonatal trials. Indeed, estimands have recently been mentioned in protocols,^35^ statistical analysis plans^36^ and results^37^ of preterm trials. When multiple births are included in a trial, researchers must decide whether the effect of the intervention on the infant or the mother is of greatest interest, as this has implications for defining the estimand, the method of analysis for outcomes measured at the infant level and ultimately, the interpretation of the trial results. This issue has recently been discussed in the context of cluster randomised trials^38^ but developing guidance specifically for trials involving both singletons and multiple births is an important area for future research.

A limitation of this systematic review is that only a subset of articles were examined by two independent reviewers, consistent with other methodological reviews.^34 39 40^ There were no discrepancies in eligibility between the trials that were examined, and, prior to being resolved, extractions only differed in total participant numbers due to postrandomisation exclusions; hence, this limitation would not be expected to change the overall findings of this paper.

In conclusion, we have identified persistent problems with the way multiple births are handled in the design, analysis and reporting of neonatal trials. We hope this methodological review will raise awareness of these ongoing issues among trialists, reviewers and journal editors, but this alone is unlikely to solve the problem. We plan to write letters to the editor regarding trial results papers and protocols that do not adequately address multiple births to further raise awareness and, in the case of protocols, provide an opportunity for trialists to make changes prior to trial completion. Clear guidance and example code for common software packages are needed to support researchers who design and analyse neonatal trials to appropriately account for clustering due to multiple, births and this work is currently in progress.

## Supplementary material

10.1136/archdischild-2024-327983 online supplemental material 1

10.1136/archdischild-2024-327983 online supplemental material 2

## Data Availability

Data are available upon reasonable request.
