# Supplementary material for: We should do better in accounting for multiple births in neonatal randomised trials: a methodological systematic review
Source: Arch Dis Child Fetal Neonatal Ed. 2024 Dec 9;110(4):e327983. doi: 10.1136/archdischild-2024-327983 (PMC12229078; doi:10.1136/archdischild-2024-327983)
Supplement: online supplemental material 1 [file fetalneonatal-110-4-s001.pdf]

Systematic Review of Multiple Births in Neonatal and Perinatal Trials: 10 years on

2023 updated protocol

28JUN23

1. Introduction

The purpose of this protocol is to describe the methods to be used in a systematic review of how multiple births are taken into account in neonatal and perinatal trials. This review will update and expand upon the previous review by Hibbs et al and Yelland et al.

Reference: Hibbs AM et al. Accounting for multiple births in neonatal and perinatal trials: systematic review and case study. The Journal of Pediatrics, 2010; 156:202-8.
Yelland, LN et al Accounting for multiple births in randomised trials: a systematic review. Archives of disease in childhood. Fetal and neonatal edition, 2015, Vol.100 (2), p.F116-F120

Note that this systematic review is not eligible for registration on PROSPERO (https://www.crd.york.ac.uk/prospero/) as this review does not have an outcome relating to the health outcomes of humans.

2. Research Questions

Design and Analysis Questions:

- Have multiple births been taken into account in the design and analysis of recent neonatal and perinatal trials?
- For trials where multiple births have been taken into account, how has this been achieved?

Design issues to consider include:
- Were multiples part of the inclusion/exclusion criteria?
- Were multiples taken into account in sample size calculations?
- Was the mother or infant randomised?
- Did the randomisation balance on multiples?
- **Is there any mention of the estimand framework?**

Analysis issues to consider include:

- Was the primary outcome defined on the mother or infant level?
- Was an analysis performed adjusting for multiple birth?
- Were subgroup analyses performed by multiple birth?
- Was an interaction test performed for treatment x multiple birth?
- Were clustered data methods used?
- Were multiples taken into account in the imputation (if performed)?

Reporting Questions:

- Has information relevant to multiple births been adequately reported in recent neonatal and perinatal trials?
- Have basic aspects of the cluster randomised trials extension to CONSORT been followed for trials where multiples were randomised to the same treatment group? (Note: limited aspects will be considered to maintain feasibility.)

Information to consider includes (* indicates relevant to cluster RCT CONSORT):

- *Were cluster RCTs identified as such in the title?
- Was it clear whether multiples were included or excluded?
- *Was it clear whether all infants from the same birth were included?
- Was it clear how infants from the same birth were randomised?
- *Was it clear who received the intervention?
- Was information presented to enable assessment of balance of multiples between groups?
- *Was it clear if and how clustering due to multiples was taken into account in the sample size?
- *Was it clear if and how clustering due to multiples was taken into account in the analysis?
- *Was the ICC reported for the primary outcome?

3. Search Strategy

The search strategy will be based on the following strategy used by Yelland et al:
“(preterm or prematur*) and (multicent*or multi-cent*)”and the filters “Randomized Controlled Trial; published in the last 5 years; Humans; English; Newborn:birth-1month”.

The authors focused on premature infants because incidence of multiple births is higher in preterm populations, and on multicentre trials for feasibility and because large multicentre trials require extensive collaboration between trialists and statisticians.

4. Eligibility Criteria

The eligibility criteria will be based on the following criteria used by Yelland et al:

‘Articles were eligible for inclusion if they reported the results of the primary outcome of a multicentre randomised trial, where the primary outcome was either measured on the infant or could be attributed to the infant. Where multiple articles reported on the primary outcome for the same trial, only the first published article was included to avoid duplication of information included in the review. The primary outcome was defined as the outcome identified by the authors as primary. If multiple primary outcomes were identified by the authors, it was defined as the outcome used to determine the sample size, provided only one outcome was used to determine the sample size, otherwise it was the first outcome identified by the authors as primary that met the eligibility criteria. If no primary outcome was identified by the authors, it was defined as the first outcome used to determine the sample size that met the eligibility criteria. Articles were excluded if none of the potential primary outcomes met the eligibility criteria, they described the methods of a trial only or they reported the results of a pilot, phase I or phase II trial, a follow-up study of a trial, secondary outcomes or analyses of a trial or multiple trials, including meta-analyses. However, articles primarily reporting the results of a single eligible trial and then adding these results to an existing meta-analysis were included.’

Articles can therefore be excluded from the systematic review for the following reasons, which will be used to complete the PRISMA flow diagram for summarizing the trial selection process:

- Protocol
- Pilot/phase I/phase II trial
- Multiple trials
- Follow-up study
- Secondary results
- Non-randomised
- Single centre
- Primary outcome definition
- Subsequent article reporting same trial

5. Review Process

Titles and abstracts of all articles identified using the search strategy described above will be exported to REDCap and examined by one reviewer to assess eligibility. Articles will be classified as ineligible (with reason) or potentially eligible. Any differences in classification will be resolved by discussion between the reviewers.

The full text of all potentially eligible articles will be obtained and examined by one reviewer using a purpose-specific data extraction form in REDCap. The form will be used to check eligibility and extract the relevant data for eligible articles.

After data has been extracted by one reviewer, a 10% random sample will be taken, stratified by eligible for data extraction (half eligible and half ineligible) to be reviewed by a second independent reviewer,

and data extracted again into REDCap. The reviewer will be unaware of the previous reviewer's eligibility status and data that was extracted.
Any differences in the data extracted will be resolved by discussion between the reviewers. The amount of discrepancies will inform whether double extraction of all data is required.

6. Data Extraction

The data extraction form will be designed to capture information collected. Refer to the data extraction form for details on the information to be collected.
