# Supplementary material for: We should do better in accounting for multiple births in neonatal randomised trials: a methodological systematic review
Source: Arch Dis Child Fetal Neonatal Ed. 2024 Dec 9;110(4):e327983. doi: 10.1136/archdischild-2024-327983 (PMC12229078; doi:10.1136/archdischild-2024-327983)
Supplement: online supplemental material 2 [file fetalneonatal-110-4-s002.pdf]

We should do better in accounting for multiple births in neonatal randomized trials: A methodological review

Supplementary materials Two

Contents

1 Listing of primary outcomes of the 62 included trials 2

2 Additional details about design features in the 62 included trials 4

3 Additional details on analyses performed in the 62 included trials. 5

1 Listing of primary outcomes of the 62 included trials

Table S1: Listing of primary outcomes for the 62 included trials.

PUBMED ID (PMID)	FIRST AUTHOR	YEAR PUBLISHED	PRIMARY OUTCOME
37129654	Sun Y	2023	live birth after embryo transfer, defined as the delivery of any number of newborns at 28 weeks or more of gestation with signs of life
37075142	Hansen ML	2023	composite of death or survival with severe brain injury
36477458	Hundscheid T	2023	composite of necrotizing enterocolitis (defined as Bell's stage IIa or higher),15 moderate-to-severe bronchopulmonary dysplasia, or death as assessed at a postmenstrual age of 36 weeks
36331493	Wessel JA	2022	live birth
36322845	Bloomfield FH	2022	survival that was free from any neurodisability at a corrected age of 2 years
36183567	Oberg	2022	Peabody Development Motor Scale-2 (PDMS-2) Total Motor Quotient at 24 months CA
35970202	Katheria AC	2023	admission to the neonatal intensive care unit for predefined criteria
35809401	Uthaya S	2022	Non AT mass
35777773	Murphy VE	2022	composite of adverse perinatal events (preterm birth, small for gestational age (SGA), perinatal mortality or neonatal hospitalisation
35467744	Zhu X	2022	total duration of IMV, need for reintubation, and ventilator-free days
35358769	Welch MG	2022	EEG power in the frontal polar region at 39-41 weeks gestational age
35320643	Watterberg KL	2022	survival without moderate or severe bronchopulmonary dysplasia at 36 weeks
35298917	van Dijk MM	2022	live birth, defined as the birth of a living child beyond 24 weeks of gestation
35123930	Groussolles M	2022	composite of adverse neonatal outcomes, namely peripartum or neonatal death or significant neonatal morbidity before hospital discharge, defined as at least 1 of the following complications: bronchopulmonary dysplasia, intraventricular hemorrhage grade III to IV, periventricular leukomalacia, necrotizing enterocolitis grade II or higher, culture-proven sepsis, and retinopathy requiring treatment
34959975	Bortolus R	2021	congenital malformations (CMs)
34902013	Dargaville PA	2021	composite of death prior to 36 weeks' postmenstrual age or physiological bronchopulmonary dysplasia19 assessed at 36 weeks' postmenstrual age
34895510	Robledo KP	2022	death or disability at 2 years
34856583	Pacagnella RC	2022	neonatal mortality and morbidity at 37 weeks
34302924	van Zanten HA	2021	median proportion of inflations during manual PPV (face mask or intubated) within the target range
34219633	Norman JE	2021	neonatal primary outcome was a composite of adverse outcomes, including stillbirth or neonatal death, periventricular leukomalacia, early respiratory morbidity (defined as any need for supplemental oxygen > 30%, continuous positive airway pressure or intratracheal ventilation or surfactant replacement therapy within the first week of life),

			intraventricular haemorrhage, necrotising enterocolitis or proven sepsis, all measured up to 28 days after the expected date of delivery.
34129204	Pan R	2021	reintubation rate of infants within 72 h of age
33793794	Rikken JFW	2021	conception followed by live birth
33627329	Dempsey EM	2021	Survival to 36 weeks of PMA without severe brain injury.
33597230	Cavallin F	2021	Proportion of normothermia at NICU admission (axillary temperature 36.5°C-37.5°C).
33577770	Beardsall K	2021	proportion of time sensor glucose concentration was 2·6-10 mmol/L for the first week of life
33523106	Hellstr	2021	severe ROP
33507929	Harding JE	2021	NICU admission
33382931	Kirpalani H	2020	composite of death or neurodevelopmental impairment
33307111	Roz	2021	Survival without cerebral palsy 24months
33248135	Stirnemann J	2021	survival at 6 months without severe neurologic morbidity
33214331	Uchiyama A	2020	treatment failure
33095526	WHO ACTION trial Collaborators	2020	primary outcomes were neonatal death alone, stillbirth or neonatal death
32898578	Hei M	2021	Los
32888085	Dani C	2021	closure rate of hsPDA
32780138	Franz AR	2020	death or disability at 2 years
32687801	Vento G	2021	mechanical ventilation in the first 72 h of life
32662862	Marc I	2020	bronchopulmonary dysplasia-free survival in infants at 36 weeks' postmenstrual age
32598909	Rehal A	2021	spontaneous birth between 24+0 and 33+6 weeks' gestation
32592693	Roman A	2020	incidence of preterm birth at <34 weeks of gestation
32407710	Tarnow-Mordi WO	2020	survival to hospital discharge or major morbidity
32237024	Wolf HT	2020	moderate to severe CP
32023373	van Kempen AAMW	2020	psychomotor development at 18 months
31940698	Juul SE	2020	Survival and disability free
31597020	Dorling J	2019	survival without moderate or severe neurodevelopmental disability at 24 months.
31523039	Crowther CA	2019	death or neurosensory disability at age 2 years
31522845	Stahl A	2019	Survival without active rop
31485014	Jain K	2019	composite of death or bronchopulmonary dysplasia (BPD) at 36 weeks postmenstrual age (PMA)
31472930	Chappell LC	2019	composite of perinatal deaths or neonatal unit admission up to infant hospital discharge
31393072	Yagui AC	2019	need for endotracheal intubation

31378395	Chappell LC	2019	composite of perinatal death (in-utero fetal death after randomisation or known neonatal death up to 7 days after birth), preterm delivery (<37 weeks' gestation), or neonatal unit admission for at least 4 h
30964778	Sun H	2020	composite of mortality or Type 1 ROP, bronchopulmonary dysplasia (BPD), serum VA levels, signs of VA toxicity, vomiting, and increased intracranial pressure
30912836	Kirpalani H	2019	BPD or death at 36 weeks' postmenstrual age
30907987	Dhillon-Smith RK	2019	live birth after at least 34 weeks of gestation
30817764	Song D	2019	number of days from initiation of oral feeds to successful FOF by bottle or breastfeeding
30721941	Hambidge KM	2019	length-for-age z score
30716790	Rong Z	2019	intubation and MV within 72 hours of life
30694322	Onland W	2019	composite of death or BPD assessed at 36 weeks' postmenstrual age.
30613992	Perrem L	2019	mean difference in time (days) to regain birth weight.
30337189	Shankaran S	2019	length of hospital stay from birth to discharge home
30322724	Subtil D	2018	Composite of late miscarriage and pre term birth
30177350	Anderson PJ	2018	academic functioning 24 months post intervention
30039171	Jensen CF	2018	weight gain velocity from randomization to postmenstrual age 40 weeks

2 Additional details about design features in the 62 included trials

For the 54/62 that did not account for clustering in the sample size, no reason for this was given in any of these trials. In those eight that did account for clustering, the details of how they achieved this were inconsistently and often incompletely reported. One trial simply stated that the sample was inflated to "to account for the impact of twins" [1]. Two trials reported the expected prevalence of multiple births (reporting prevalence of 25%, [2,3]), and two reported the assumed average cluster size (1.25 [3] and 2 [4]). Four trials stated the assumed value of the intraclass correlation coefficient (ICC) as a measure of the degree of expected similarity of outcomes of infants from the same birth, which ranged between 0.3 and 0.9 [2–5], however only two provided a reference for this value [2,5]. Five trials explicitly reported a design effect that fell in the range of 1.1-1.2 [2,3,6–8], indicating the required sample size was 10-20% larger than a trial including singletons only.

3 Additional details on analyses performed in the 62 included trials.

When clustering was taken into account (35%; 22/62), this was more commonly in the primary analysis (18/22) rather than in a secondary or sensitivity analyses (4/22). Accounting for clustering was performed by generalized estimating equations (GEE) in 55% (12/22)[3,6–16], mixed models in 27% (6/22) [1,2,4,17–19], both GEE and mixed models in one trial [5], modified Poisson or logistic regression with robust standard error estimates in two trials [20,21], and one trial stated in the supplementary material that the analysis model included “a clustering feature for multiple births” [22]. In those trials that performed analyses by GEE (n=13), only three reported the working correlation structure used (all reported an exchangeable working correlation structure) [6,10,12]. All trials that accounted for clustering in the sample size calculations also accounted for clustering in the primary analysis.

Multiple imputation was performed in 11% of trials (7/62)[3,9,10,12,22–24], most commonly by chained equations (6/7). Only two trials [9,10] reported using multiple births (singleton versus multiple birth) as a fixed covariate in the imputation models followed by a mixed model [9] or GEE [10] analysis of the imputed datasets, taking into account the clustering of outcomes.

References

[1] Øberg GK, Handegård BH, Campbell SK, Ustad T, Fjørtoft T, Kaaresen PI, et al. Two-year motor outcomes associated with the dose of NICU based physical therapy: The noppi RCT. Early Hum Dev 2022;174:105680. https://doi.org/10.1016/j.earlhumdev.2022.105680.

[2] Dorling J, Abbott J, Berrington J, Bosiak B, Bowler U, Boyle E, et al. Controlled trial of two incremental milk-feeding rates in preterm infants. N Engl J Med 2019;381:1434–43. https://doi.org/10.1056/NEJMoa1816654.

[3] Juul SE, Comstock BA, Wadhawan R, Mayock DE, Courtney SE, Robinson T, et al. A randomized trial of erythropoietin for neuroprotection in preterm infants. N Engl J Med 2020;382:233–43. https://doi.org/10.1056/NEJMoa1907423.

[4] Jensen CF, Sellmer A, Ebbesen F, Cipliene R, Johansen A, Hansen RM, et al. Sudden vs pressure wean from nasal continuous positive airway pressure in infants born before 32 weeks of gestation: A randomized clinical trial. JAMA Pediatr 2018;172:824–31. https://doi.org/10.1001/jamapediatrics.2018.2074.

[5] Stirnemann J, Slaghekke F, Khalek N, Winer N, Johnson A, Lewi L, et al. Intrauterine fetoscopic laser surgery versus expectant management in stage 1 twin-to-twin transfusion syndrome: An international randomized trial. Am J Obstet Gynecol 2021;224:528.e1–12. https://doi.org/10.1016/j.ajog.2020.11.031.

[6] Crowther CA, Ashwood P, Andersen CC, Middleton PF, Tran T, Doyle LW, et al. Maternal intramuscular dexamethasone versus betamethasone before preterm birth (ASTEROID): A multicentre, double-blind, randomised controlled trial. Lancet Child Adolesc Health 2019;3:769–80. https://doi.org/10.1016/s2352-4642(19)30292-5.

[7] Kirpalani H, Ratcliffe SJ, Keszler M, Davis PG, Foglia EE, Te Pas A, et al. Effect of sustained inflations vs intermittent positive pressure ventilation on bronchopulmonary dysplasia or death among extremely preterm infants: The SAIL randomized clinical trial. Jama 2019;321:1165–75. https://doi.org/10.1001/jama.2019.1660.

[8] Marc I, Piedboeuf B, Lacaze-Masmonteil T, Fraser W, Mâsse B, Mohamed I, et al. Effect of maternal docosahexaenoic acid supplementation on bronchopulmonary dysplasia-free survival in breastfed preterm infants: A randomized clinical trial. Jama 2020;324:157–67. https://doi.org/10.1001/jama.2020.8896.

[9] Hansen ML, Pellicer A, Hyttel-Sørensen S, Ergenekon E, Szczapa T, Hagmann C, et al. Cerebral oximetry monitoring in extremely preterm infants. N Engl J Med 2023;388:1501–11. https://doi.org/10.1056/NEJMoa2207554.

[10] Robledo KP, Tarnow-Mordi WO, Rieger I, Suresh P, Martin A, Yeung C, et al. Effects of delayed versus immediate umbilical cord clamping in reducing death or major disability at 2 years corrected age among very preterm infants (APTS): A multicentre, randomised clinical trial. Lancet Child Adolesc Health 2022;6:150–7. https://doi.org/10.1016/s2352-4642(21)00373-4.

[11] Pacagnella RC, Silva TV, Cecatti JG, Passini Jr R., Fanton TF, Borovac-Pinheiro A, et al. Pessary plus progesterone to prevent preterm birth in women with short cervixes: A randomized controlled trial. Obstet Gynecol 2022;139:41–51. https://doi.org/10.1097/aog.0000000000004634.

[12] Rozé JC, Cambonie G, Le Thuaut A, Debillon T, Ligi I, Gascoin G, et al. Effect of early targeted treatment of ductus arteriosus with ibuprofen on survival without cerebral palsy at 2 years in infants with extreme prematurity: A randomized clinical trial. J Pediatr 2021;233:33–42.e2. https://doi.org/10.1016/j.jpeds.2020.12.008.

[13] Tarnow-Mordi WO, Abdel-Latif ME, Martin A, Pammi M, Robledo K, Manzoni P, et al. The effect of lactoferrin supplementation on death or major morbidity in very low birthweight infants (LIFT): A multicentre, double-blind, randomised controlled trial. Lancet Child Adolesc Health 2020;4:444–54. https://doi.org/10.1016/s2352-4642(20)30093-6.

[14] Wolf HT, Brok J, Henriksen TB, Greisen G, Salvig JD, Pryds O, et al. Antenatal magnesium sulphate for the prevention of cerebral palsy in infants born preterm: A double-blind, randomised, placebo-controlled, multi-centre trial. Bjog 2020;127:1217–25. https://doi.org/10.1111/1471-0528.16239.

[15] Song D, Jegatheesan P, Nafday S, Ahmad KA, Nedrelow J, Wearden M, et al. Patterned frequency-modulated oral stimulation in preterm infants: A multicenter randomized controlled trial. PLoS One 2019;14:e0212675. https://doi.org/10.1371/journal.pone.0212675.

[16] Onland W, Cools F, Kroon A, Rademaker K, Merkus MP, Dijk PH, et al. Effect of hydrocortisone therapy initiated 7 to 14 days after birth on mortality or bronchopulmonary dysplasia among very preterm infants receiving mechanical ventilation: A randomized clinical trial. Jama 2019;321:354–63. https://doi.org/10.1001/jama.2018.21443.

[17] Harding JE, Hegarty JE, Crowther CA, Edlin RP, Gamble GD, Alsweiler JM. Evaluation of oral dextrose gel for prevention of neonatal hypoglycemia (hPOD): A multicenter, double-blind randomized controlled trial. PLoS Med 2021;18:e1003411. https://doi.org/10.1371/journal.pmed.1003411.

[18] Chappell LC, Brocklehurst P, Green ME, Hunter R, Hardy P, Juszczak E, et al. Planned early delivery or expectant management for late preterm pre-eclampsia (PHOENIX): A randomised controlled trial. Lancet 2019;394:1181–90. https://doi.org/10.1016/s0140-6736(19)31963-4.

[19] Chappell LC, Bell JL, Smith A, Linsell L, Juszczak E, Dixon PH, et al. Ursodeoxycholic acid versus placebo in women with intrahepatic cholestasis of pregnancy (PITCHES): A randomised controlled trial. Lancet 2019;394:849–60. https://doi.org/10.1016/s0140-6736(19)31270-x.

[20] Bloomfield FH, Jiang Y, Harding JE, Crowther CA, Cormack BE. Early amino acids in extremely preterm infants and neurodisability at 2 years. N Engl J Med 2022;387:1661–72. https://doi.org/10.1056/NEJMoa2204886.

[21] Norman JE, Norrie J, MacLennan G, Cooper D, Whyte S, Chowdhry S, et al. The arabin pessary to prevent preterm birth in women with a twin pregnancy and a short cervix: The STOPPIT 2 RCT 2021;25:44. https://doi.org/10.3310/hta25440.

[22] Oladapo OT, Vogel JP, Piaggio G, Nguyen MH, Althabe F, Gülmezoglu AM, et al. Antenatal dexamethasone for early preterm birth in low-resource countries. N Engl J Med 2020;383:2514–25. https://doi.org/10.1056/NEJMoa2022398.

[23] Groussolles M, Winer N, Sentilhes L, Biquart F, Massoud M, Vivanti AJ, et al. Arabin pessary to prevent adverse perinatal outcomes in twin pregnancies with a short cervix: A multicenter randomized controlled trial (PESSARONE). Am J Obstet Gynecol 2022;227:271.e1–13. https://doi.org/10.1016/j.ajog.2022.01.038.

[24] Kempen A van, Eskes PF, Nuytemans D, Lee JH van der, Dijksman LM, Veenendaal NR van, et al. Lower versus traditional treatment threshold for neonatal hypoglycemia. N Engl J Med 2020;382:534–44. https://doi.org/10.1056/NEJMoa1905593.
